# Development of A Sensitive and Specific Epitope-Blocking ELISA for Universal Detection of Antibodies to Human Enterovirus 71 Strains

**DOI:** 10.1371/journal.pone.0055517

**Published:** 2013-01-31

**Authors:** Fang He, Tanja K. Kiener, Xiao Fang Lim, Yunrui Tan, Kattur Venkatachalam Ashok Raj, Manli Tang, Vincent T. K. Chow, Qingfeng Chen, Jimmy Kwang

**Affiliations:** 1 Animal Health Biotechnology, Temasek Life Sciences Laboratory, Singapore, Singapore; 2 Department of Microbiology Faculty of Medicine, National University of Singapore, Singapore, Singapore; 3 Humanized Mouse Unit, Institute of Molecular and Cell Biology, Singapore, Singapore; 4 Infectious Diseases, The Singapore-Massachusetts Institute of Technology Alliance for Research and Technology, Singapore, Singapore; University of Arizona, United States of America

## Abstract

**Background:**

Human Enterovirus 71 (EV71) is a common cause of hand, foot and mouth disease (HFMD) in young children. It is often associated with severe neurological diseases and mortalities in recent outbreaks across the Asia Pacific region. Currently, there is no efficient universal antibody test available to detect EV71 infections.

**Methodology/Principal Finding:**

In the present study, an epitope-blocking ELISA was developed to detect specific antibodies to human EV71 viruses in human or animal sera. The assay relies on a novel monoclonal antibody (Mab 1C6) that specifically binds to capsid proteins in whole EV71 viruses without any cross reaction to any EV71 capsid protein expressed alone. The sensitivity and specificity of the epitope-blocking ELISA for EV71 was evaluated and compared to microneutralization using immunized animal sera to multiple virus genotypes of EV71 and coxsackieviruses. Further, 200 serum sample from human individuals who were potentially infected with EV71 viruses were tested in both the blocking ELISA and microneutralization. Results indicated that antibodies to EV71 were readily detected in immunized animals or human sera by the epitope blocking ELISA whereas specimens with antibodies to other enteroviruses yielded negative results. This assay is not only simpler to perform but also shows higher sensitivity and specificity as compared to microneutralization.

**Conclusion:**

The epitope-blocking ELISA based on a unique Mab 1C6 provided highly sensitive and 100% specific detection of antibodies to human EV71 viruses in human sera.

## Introduction

Over the last decade, frequent epidemic outbreaks of hand, foot and mouth disease (HFMD) in young children below 6 years old have been observed in the Asia-Pacific region. HFMD is caused by different etiological agents from the enterovirus family, mainly Coxsackievirus A16 and Enterovirus 71 from the human enterovirus A family [1]. EV71 (BrCr strain) was first isolated and identified in the United States in 1969 [2], and was not associated with hand, foot mouth disease (HFMD) until 1973, when small epidemics broke out in Japan and Sweden [3], [4]. From then on, successive waves of EV71 outbreaks have been reported globally, in the United Kingdom, Australia, Sweden, Bulgaria, Japan, China, Hong Kong, Taiwan, Malaysia and Singapore [3], [5], [6], [7], [8], [9], [10], [11]. Severe disease and neurological complications are more often associated with EV71 infection, and can occasionally lead to fatal brain stem encephalitis in young children. EV71 has been responsible for fatal cases of HFMD during the large outbreaks in Malaysia in 1997 [12], Taiwan in 1998, 2000 and 2001 [11], [13], Australia in 1999 [14], [15], Singapore in 2000 [14], [16] and China in 2008. From 1999 to 2010, HFMD outbreaks caused by EV71 have affected more than 500,000 children and resulted in more than 200 deaths in China. In fact, after the eradication of poliovirus, EV71 is now regarded as the most important neurotropic enterovirus and a threat to global public health [16], [17], [18], [19]. The rapid progression and high mortality of severe hand, foot and mouth disease makes the direct detection of EV71 early in infection essential.

The genome of enteroviruses encodes a single large polyprotein that consists of structural region P1 and non-structural regions P2 and P3. P1 can be processed by virus-encoded proteinase, which results in viral capsid subunit proteins VP0, VP1 and VP3. For some enteroviruses, such as poliovirus, VP0 might be cleaved further to yield VP2 and VP4 [20]. Like poliovirus, EV71 is a small, nonenveloped, positive-stranded RNA viral pathogen within the Picornavirus family. The genome of EV71 contains a single large coding region flanked by 59- and 39- untranslated regions (59- and 39 - UTR). The coding region is translated to a single polypeptide, which is then processed by viral proteases to yield nonstructural proteins and 4 capsid proteins: VP1, VP2, VP3 and VP4 assembled as pentameric subunits [21]. These capsid proteins form the icosahedral structure, with VP1-3 exposed on the virus surface and VP4 arranged internally [22]. Capsid proteins are thought to play an important role in immunogenicity, viral pathogenesis and virulence [23]. Based on the VP1 gene sequence, EV71 is divided into three major genogroups (denoted A, B and C), and various subgenogroups within genogroups B (B1 to B5) and C (C1 to C5) [24]. VP1, involved in the recognition of EV71 receptors, displays major immunogenicity. Besides, neutralizing or antigenic epitopes on the VP0 and VP2 proteins have been described in other members of the picornavirus family including poliovirus [25], [26], coxsackievirus A9 [27], foot-mouth-disease virus [28], and parechovirus [29]. Furthermore, VP0 has been proposed as a diagnostic tool to detect anti-human parechovirus 1 antibodies in patient sera [30], [31].

Serological investigations to detect specific antibodies from EV71 infection or vaccination in humans are critical to the success of disease prevention and control programs. However, due to the lack of a specific and sensitive monoclonal antibody, there are no many serologic tests available against EV71. Microneutralization is currently used as the major antibody test for EV71. However, the test is labor-intensive and its sensitivity is limited, rendering it impractical for rapid and high-throughput diagnostics [32]. Indirect ELISA has been widely used in serologic surveillance against viral pathogens. However, cross-reacting antibodies elicited by infection or vaccination with non-EV71 enterovirus can yield false positive test results that reduce the value of indirect EV71 ELISA in humans [33].

In this study, an epitope-blocking ELISA for EV71 antibody detection was developed based on a unique Mab 1C6. 1C6 can specifically bind to capsid proteins in whole EV71 viruses without any cross reaction to individual EV71 capsid proteins. 1C6 can detect EV71 infection in mammalian cells and virus-like-particle expression in insect cells, while no signals were detected in cells expressing single EV71 capsid protein. The unique recognition of native capsid proteins of EV71 allows 1C6 to efficiently compete with other antibodies against native EV71 viruses. This assay yields a positive result when antibodies to EV71 capsid proteins in test sera block binding of labeled 1C6 to EV71. In practice, the color intensity of the reaction resulting from antigen bound Mab is inversely proportional to the amount of epitope specific antibody present in test serum. The results of this study indicated that the epitope blocking ELISA (EB-ELISA) with Mab 1C6 provided improved sensitivity compared to virus neutralization and 100% specific detection of antibodies to EV71 viruses in human sera.

## Materials and Methods

### Ethics Statement

All animal experiments were carried out in accordance with the guidelines for Animal Experiments of the National Institute of Infectious Disease (NIID).

Experiment protocols were reviewed and approved by Institutional Animal Care and Use Committee of the Temasek Life Sciences Laboratory, National University of Singapore, Singapore (IACUC number TLL-11-010).

All the protocols and usage involving human blood samples were approved by the Institutional Review Board of the National University of Singapore. Informed written consent was obtained from the next of kin, carers or guardians on the behalf of the minors/children participants before the study was conducted.

### Viruses and cells

Wild-type enterovirus 71 and coxsackievirus strains were received from the Human Genome Laboratory, Department of Microbiology, Yong Loo Lin School of Medicine, National University of Singapore, Singapore. Additionally, the virus EV71-C4-Fuyang (NCBI accession #EU703813.1) has been constructed in the lab by reverse genetics [34]. All virus strains have been propagated in rhabdomyosarcoma (RD) cells grown in DMEM medium (Gibco) with 10% fetal bovine serum (FBS). Virus was added to the culture medium, incubated at 37°C for 48 h when over 90% of cytopathic effect (CPE) was observed. The supernatant was collected and the virus activity was tested on RD cells in an end-point dilution assay (Reed & Muench, 1938) to calculate the tissue culture infective dose (TCID50). Before further experimentation, virus was inactivated with BEI. A 0.2 M solution of 2-bromoethylamine hydrobromide in 0.4 M NaOH (BEI) was prepared and incubated overnight at room temperature. BEI was then added to the cell supernatant containing virus and incubated for 48 h at 37°C. BEI was neutralized with 1/10 total volume of 1 M sodium thiosulfate. Cell supernatant was then clarified by centrifugation at 7,500 g for 30 min and filtration through a 0.2 um cut-off filter (Millipore). The virus was concentrated 20 times by an ultraspin at 100,000 g for 3 h and re-suspended in PBS.

### Production and Characterization Mab 1C6

SPF BALB/c mice were immunized with inactivated virus strain NUH0083-B5 in 0.1 ml of PBS, emulsified with adjuvant (Seppic, France) at a 1∶1 ratio. Mice were subjected to two boosters at 14-days intervals. Mice were euthanized three days after being subjected to a final intraperitoneal booster, and. spleen cells were harvested and fused with SP2/0 myeloma cells as described previously [35]. Hybridoma cells were subjected to screening by Immunofluorescence assay (IFA), and positive clones secreting EV71-specific monoclonal antibodies were subcloned and cultured. Immunoglobulins from selected Mabs were isotyped using a commercial kit (Amersham Bioscience, England). Mouse Mab 1C6 was covalently conjugated to horseradish peroxidase (HRP) and purified from unbound enzyme using a commercial kit (Roche).

### Experimental serum samples

Groups of Guinea pigs (n = 4) were injected intramuscularly with different inactivated EV71 viruses (Table 1) emulsified in adjuvant (SEPPIC, France). The injections were repeated twice at two-week intervals. In addition, groups of mice (n = 4) were immunized with coxsackievirus A4, A6, A10 and A16 individually. Blood was collected 14 days after the 2nd immunization.

**Table 1:** EV71 viruses used in this study.

Name	Accession number	Subgenotypes
**BrCr**	U22521	A
**RG EV71-VP1(B1)**	AF135901	B1
**7423/MS/87**	ETU22522	B2
**RG EV71-VP1(B3)**	AF376093	B3
**HFM41**	AF316321	B4
**NUH0083**	FJ461781	B5
**557-VP1(B5)**	HQ285105	B5
**Y90-3761**	AB433864	C1
**NUH0075**	FJ172159	C2
**RG EV71-VP1(C3)**	AY125973	C3
**75-Yamagata**	AB177813	C4
**3437/SIN/06**	GU222654	C5

All animal experiments were carried out in accordance with the guides for Animal Experiments of the National Institute of Infectious Disease (NIID). Experiment protocols were reviewed and approved by Institutional Animal Care and Use Committee of the Temasek Life Sciences Laboratory, National University of Singapore, Singapore.

### Human serum panels

400 human serum samples were collected from Singapore citizens and permanent residents who were ethnic Chinese, Malay and Indian aged between 1–17 years attending inpatient services or day surgery and provided by Department of Microbiology, Faculty of Medicine, National University of Singapore. Patients were excluded if they were known to be immunocompromised, on immunosupressive therapy, or had been diagnosed with measles, mumps, rubella, chickenpox, diphtheria, pertussis, poliomyelitis, hepatitis B, dengue or HFMD. Human umbilical cord blood samples were obtained from Singapore Cord Blood Bank. Approval was obtained by the Institutional Review Board, National University of Singapore.

### Indirect immunofluoresence assay

African green monkey kidney cells (Vero cells) were used for IFA. Cells were seeded overnight onto 96-well microtiter plates and infected with EV71. Upon observation of CPE after 48 h at 37°C, cells were fixed with 4% paraformaldehyde (pH7.4) for 20 min and permeabilized with 0.1% Triton-X/PBS for 5 min. Cells were blocked with 5% FBS/PBS for 30 min, washed and incubated in hybridoma cell supernatant or primary antibody solution for 1 h followed by incubation in FITC-coupled secondary antibodies for 1 h in room temperature. Cells were washed in 0.1% Tween/PBS for thrice for 5 min between each step. Results were documented with an inverted microscope (Olympus) with Nikon ACT-1 software.

### Microneutralization

Neutralization activity of serum samples were determined by in vitro microneutralization assay in RD cells. Two-fold serial monoclonal antibody dilutions (50 µl each) were mixed with equal volume of 200 TCID_50_ of virus, and incubated at 37°C for 1 h. The antibody-virus mixtures were then added to the wells of the microtiter plates containing RD cells. The highest dilution of serum samples that inhibited virus growth was considered as the neutralization antibody titer and was determined after incubation at 37°C for 96 h. Serum samples were heated at 56°C for 30 min to inactivate complements before use. Each assay was performed independently for three times.

### Epitope-Blocking ELISA

Optimal dilutions of purified EV71 viral antigen and Mab were determined by checkerboard titration to yield sub-saturating levels of the Mab. U-bottomed 96-well ELISA plates were coated with purified EV71 virus (B4 5865/SIN/09 and B5 NUH0083/SIN/09) prepared as described above (500 ng/well) and incubated overnight at 4°C in coating buffer (0.1 mol/L carbonate/bicarbonate, pH 9.6). Antigen-coated plates were washed with PBS (pH 7.5) containing 0.05% Tween 20 (PBST) and nonspecific sites were blocked with 100 mL blocking buffer (PBST containing 5% skim milk) for 40 min at 37°C. Test serum samples were serially diluted two fold in PBST, and 100 uL was added to each well and incubated for 1 h at 37°C. The wells were rinsed four times with PBST and incubated with 120 ng of HRP conjugated Mab 1C6 in 100 uL PBST with 1% skim milk for 1 h at 37°C. The wells were rinsed with PBST and incubated with 100 uL of 3, 39, 5, 59-tetramethyl benzidine (TMB, Sigma, USA). The reaction was stopped by adding 0.1N Sulfuric acid and the optical density (OD) determined at 450 nm using a multiwell plate reader. The OD intensity reduction caused by serum antibodies blocking Mab binding was calculated for each sample dilution by using the formula: % inhibition = [(negative reference serum OD-test serum OD)/(negative reference serum OD-positive reference serum OD)]×100%. To determine the cut-off value, specific pathogen-free mice sera were obtained from the Animal Health Biotechnology Serum Bank, Temasek Life Sciences Laboratory, Singapore.

## Results

### Characterization of Mab 1C6

**Figure 1:**
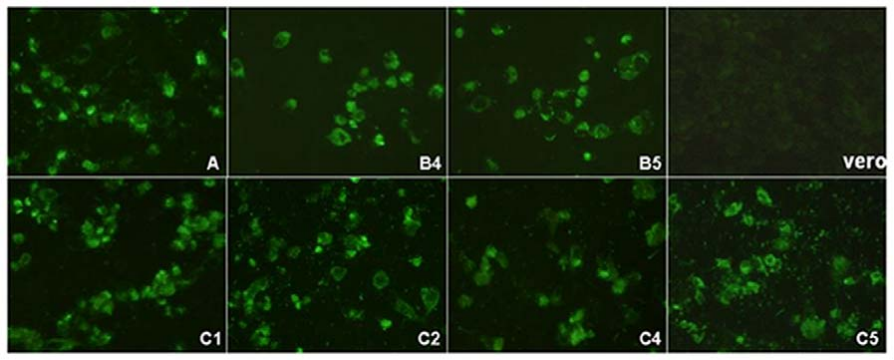
Mab 1C6 detects variable EV71 strains. IFA of Vero cells individually infected with different EV71 strains. Cells were labeled with Mab 1C6 followed by anti-mouse FITC secondary antibody.

**Figure 2:**
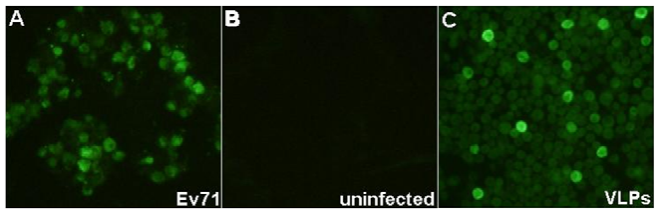
Characterization of Mab 1C6. Cells were labeled with Mab 1C6 followed by anti-mouse FITC secondary antibody. (A) Vero cells infected with EV71-B5 (NUH0083). (B) Non-infected Vero cells. (C) Insect cells expressing virus-like particles (VLPs) of EV71.

Mab 1C6 belongs to IgG2a, as identified with an isotyping Kit. 1C6 could detect EV71 viral protein expression in infected RD cells as shown by IFA. No signal was detected in non-infected RD cells, confirming that this is an EV71-specific Mab. IFA with EV71 of different genotypes was performed, indicating that 1C6 is able to react with all the genotypes tested in the study. Figure 1 showed the representative results with 1C6 to detect EV71 expression in infected RD cells. 1C6 was tested to be negative in Western blot against EV71 whole virus lysate, indicating that it recognizes a conformational epitope instead of a linear epitope. Attempts were made to assign the epitope to a specific EV71 capsid protein using recombinant baculovirus that express VP0, VP1, VP2, VP3, or VP4 in insect cells, but no signal was detected with 1C6 against any individual capsid protein. However, 1C6 could detect virus-like particles of EV71 generated in insect cells co-infected with VP0, VP1, and VP3 proving that 1C6 is specific for the EV71 capsid (Fig. 2). No neutralizing activity was observed for 1C6 in EV71 virus neutralization test.

### Development of blocking ELISA with 1C6

Serum antibodies to capsid proteins of EV71 can be detected by virtue of their ability to block the binding of a specific Mab to the target epitope in an ELISA assay. To develop this assay, serum panels from normal and EV71-immunized mice or guinea pigs were used (Table 2). First, a panel of 20 normal mouse and 5 guinea pig serum samples lacking antibodies to EV71 was used to determine the baseline of non-specific reduction in 1C6 binding to capsid antigen in the EB-ELISA. Mean reduction of EB-ELISA readings (i.e. blocking) was 5.9% for this serum panel, with a standard deviation (SD) of 6.3. Specific blocking activities can be determined with 95% confidence if a “cut-off value” of ≥30% is set for serum samples. The latter was obtained by adding 3 SD to the mean 5.9% blocking (5.9+18.9 = 24.8%). In the test, the dilution factor of each serum sample at was recorded when it presented ≥30% signal blocking rate. Additionally, the blocking rate of each sample diluted at 20 times was recorded for comparison.

**Table 2:** The summary of serum samples used in the study.

Species	Quantity	Description
Humans	100	Virus neutralization titer ≥8
Humans	100	Virus neutralization titer <8
Guinea pig	48	EV71 immunized, 4 per group.
Mouse	16	Coxsackievirus immunized, 4 per group.
Guinea pig	5	Uninoculated
Mouse	20	Uninoculated

### Specificity of the blocking ELISA with 1C6

**Figure 3:**
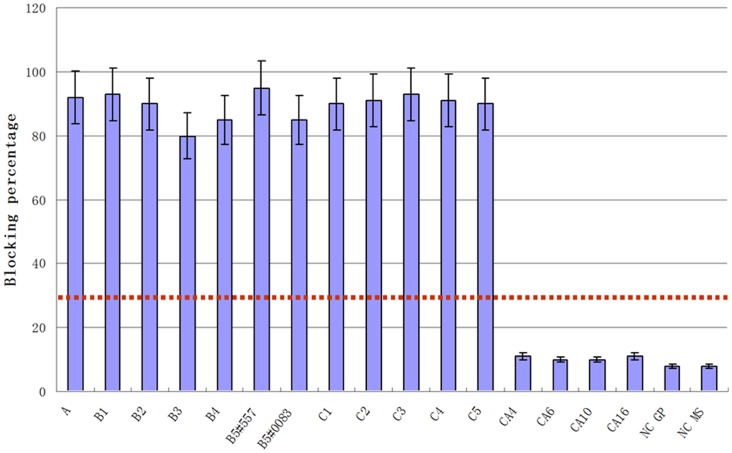
Sensitivity and specificity of the EB-ELISA. Performance of the EB-ELISA with sera from guinea pigs immunized with different strains of EV71 or mice with different coxsackievirus. Sera were collected 14 days after the 2nd immunization and normalized to a virus neutralization titer of 16 before the EB-ELISA. Inhibition above the cut-off value of 30% blocking was considered as positive; i.e. antibodies to EV71 were present. The results were expressed as the arithmetic mean of percent blocking values (n = 4/group, whiskers above bars represent the standard error of the mean). NS: Normal preimmune serum; GP: guinea pig; MS: mouse. Dotted line: cutoff values.

The specificity of the blocking ELISA with Mab 1C6 was investigated using a panel of antisera from experimentally immunized guinea pigs. Sera collected 10 days after the second immunization were first diluted to obtain neutralization titer of 16 to the homologous virus to normalize antibody concentrations. The serum samples were further diluted 20 times prior to use. The blocking ELISA endpoint of the VN (virus neutralization)-normalized sera was then determined by analysis of log2 serial dilutions. Sera from guinea pigs immunized with different EV71 viruses (Table 1) yielded blocking values above the 30% cutoff in blocking ELISA with Mab 1C6 (Fig. 3). Most samples showed ≥80% inhibition which presented negative neutralization activity at the same dilution, indicating that the blocking ELISA detected low levels of antibody to EV71. Moreover, together with sera from non-inoculated mice and guinea pigs, sera from mice immunized individually with coxsackievirus CA4, CA6, CA10 and CA16 showed maximum blocking of, 12%, well below the 30% threshold established for samples containing specific antibodies (Fig. 3). These results indicated that the blocking ELISA could successfully differentiate serum samples containing antibodies to EV71 from those sera containing antibodies to other enterovirus subtypes.

### Sensitivity of the blocking ELISA with 1C6

**Figure 4:**
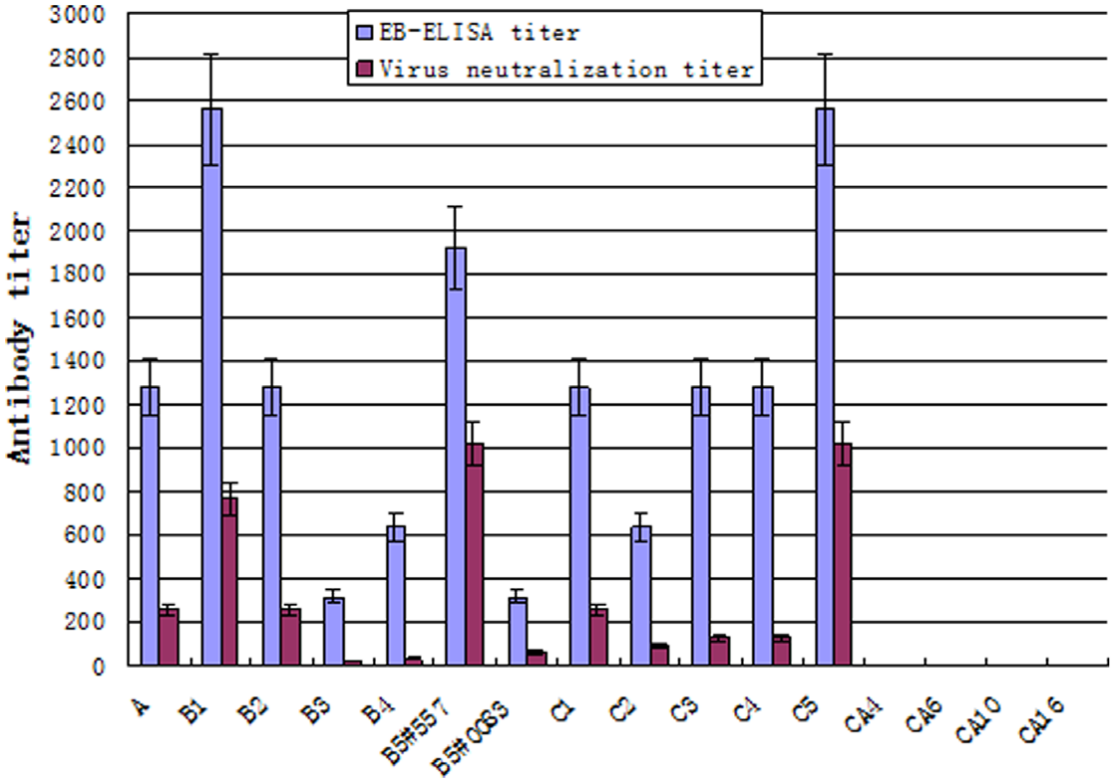
Comparison of EB-ELISA to microneutralization assay. The EV71 antibody detection sensitivity and specificity of the EB-ELISA and microneutralization assays were compared using guinea pig immune sera collected 14 days after the 2nd immunization to determine the endpoint of inhibition. The EB-ELISA titer was determined as described in the Methods section. The neutralization assays were performed using B5 NUH0083 EV71 virus. The results were expressed as the geometric mean titers (n = 4/group, whiskers above bars represent the standard error of the mean).

The sensitivity of the blocking ELISA was primarily determined by comparison to virus neutralization using a purified neutralizing monoclonal antibody 51 [36] against EV71 (Table 3). In EB-ELISA, 400 ng of Mab 51 was sufficient to reach the endpoint corresponding to a blocking rate of more than 30%, while at least 1250 ng of the same Mab 51 was needed to neutralize 100 TCID50 of EV71 virus. Additional comparisons of EB-ELISA and virus neutralization were then made using immunized guinea pig sera. As shown in Fig. 4, the neutralization titers, against B5 strain (NUH0083) of sera from guinea pigs immunized with variant EV71s individually, range from 16 to 1024. The same batch of sera was tested in the blocking ELISA where the endpoint titers range from 160 to 2560. No positive activity was detected for coxsackievirus immunized serum samples by either test. The comparison indicated that the blocking ELISA was able to detect a lower concentration of specific antibody and present a higher signal titer than virus neutralization (*p*<0.005).

**Table 3:** The detection limit of EB-ELISA and microneutralization assay.

EB-ELISA		Microneutralization	
Mab amount	Inhibition rate	Mab amount	Titer
10 ug	63.32%	5 ug	32
2 ug	45.63%	2.5 ug	16
***0.4 ug***	33.45%	***1.25 ug***	8
0.08 ug	26.83%	0.63 ug	<8
0.016 ug	13.24%	0.32 ug	<8

The detection limit of each test was indicated in bold and italics format.

### Test with human serum samples

**Figure 5:**
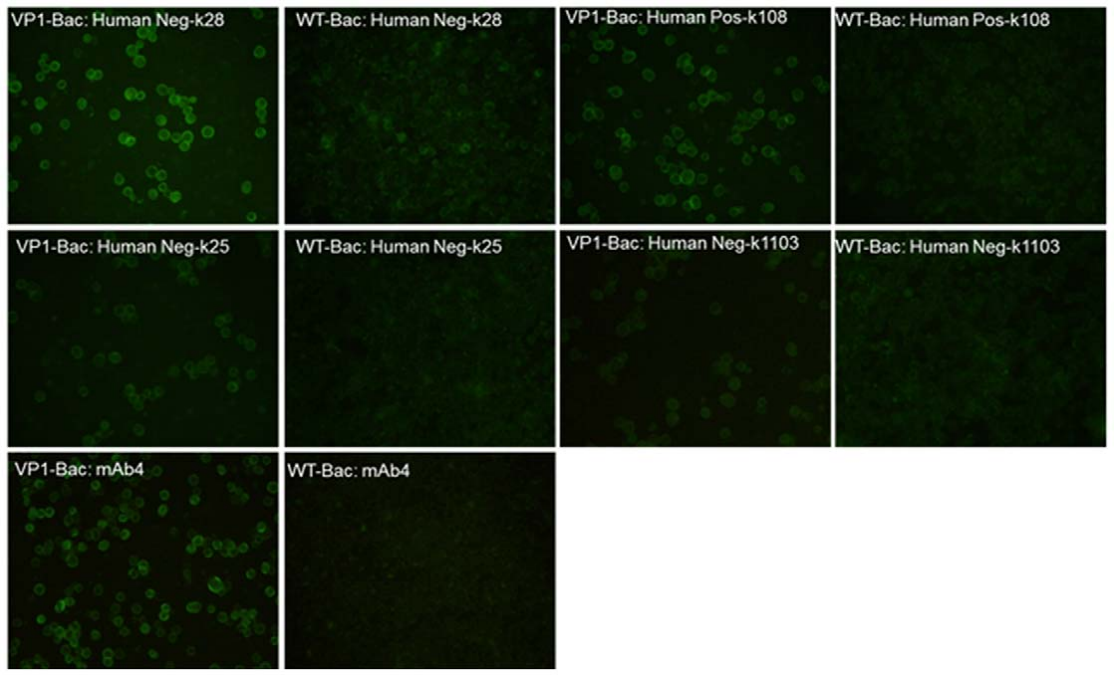
Detection of anti-VP1 antibody in human sera with baculovirus infected SF9 cells. SF9 cells were infected with either recombinant baculovirus expressing VP1 of EV71 (VP1-Bac) or baculovirus wild type (WT-Bac). Cells were fixed 36 h post infection and stained with different serum samples and secondary FITC antibody. Mab 4 is a VP1 specific monoclonal antibody that was used as a positive control. Human Neg: human serum sample which is negative in virus neutralization. Human Pos: human serum sample which is positive in virus neutralization.

To determine whether the 1C6 based blocking ELISA consistently detect antibodies elicited by natural EV71 infection in humans, the assay was further evaluated with human serum samples. 100 VN-positive serum samples with a neutralization activity of no less than 8 and 300 VN-negative serum samples (neutralization titer is less than 8) were tested in the blocking ELISA. Besides, 8 samples of human cord blood, which do not consist of a significant level of EV71 antibodies, were tested together as negative controls. Table 4 summarizes the representative results of these serum samples. The 100 samples with positive neutralization titer presented significant blocking percentages. All of these VN-positive samples, which were diluted at 20 times with PBS, were able to block more than 70% of the signals from labeled 1C6 in this blocking ELISA. Interestingly, some of those 300 samples without positive titer in neutralization showed blocking activity in the EB-ELISA as well. The blocking percentage ranged from 15% to 90% with sera diluted 20 times. With the cut-off value of 30% blocking, 249 out of 300 VN-negative samples were found to be positive in the 1C6 blocking ELISA, while all the seven cord blood samples presented negative reactivity (about 9–23% blocking) in the test. The findings were confirmed in immuno-fluorescence assays with VP1 expressing baculovirus infected SF9 cells. As shown in Figure 5, both VN-positive and VN-negative human serum samples, which were tested to be strongly positive in the EB-ELISA, clearly detected VP1 expression in recombinant baculovirus infected SF9 cells. Meanwhile, human sera with blocking percentages approaching the cut-off value of 30% were tested in the same VP1 expressing cells. Positive fluorescent signals were observed with sample K25 (blocking rate 34.42%). Although signals were weaker those from strongly positive samples, bright staining was able to be found in individual cells. Signals from K1103 (blocking rate 24.37%) were even weaker than K25. The sample K1103 is accepted to be negative in IFA as no clear and bright signal was observed in any cells. Due to the lack of a clear cut-off point, IFA was only used as a confirmative method in serology test. These results revealed that EV71 specific antibodies exist in some human samples at levels below the detection limit of virus neutralization but within the detectable range of the EB-ELISA. These findings further confirm that the blocking ELISA with 1C6 has better sensitivity than virus neutralization and a clearer cut-off point than IFA test. The study suggests that the percentage of EV71 exposed population could be higher than currently evaluated based on virus neutralization test.

**Table 4:** Analysis of human serum samples in EB-ELISA.

Clinical human serum samples	Virus neutralization titer	EB-ELISA titer at 30% cut-off	Inhibition in EB-ELISA at 1∶20 dilution
**K8**	16	640	94.27
**K9**	16	320	87.04
**K41**	32	320	89.34
**K43**	256	2560	99.86
**K52**	32	160	93.60
**K99**	128	1280	99.85
**K108**	32	640	98.30
**K146**	64	640	99.95
**K148**	64	640	93.93
**K198**	64	640	99.92
**K200**	32	1280	99.57
**K251**	32	1280	99.86
**K289**	16	320	93.27
**K471**	32	1280	99.63
**K508**	256	2560	98.67
**K511**	32	160	79.84

Clinical human serum samples	Virus neutralization titer	EB-ELISA titer at 30% cut-off	Inhibition in EB-ELISA at 1∶20 dilution
**K1**	<8	160	48.36
**K2**	<8	160	86.88
**K25**	<8	40	34.42
**K26**	<8	80	66.72
**K28**	<8	80	48.36
**K36**	<8	320	87.70
**K39**	<8	40	34.42
**K58**	<8	160	69.67
**K85**	<8	160	45.24
**K87**	<8	<20	22.95
**K97**	<8	160	70.98
**K100**	<8	80	64.59
**K116**	<8	160	40.02
**K117**	<8	80	59.67
**K135**	<8	160	43.44
**K140**	<8	40	37.7
**K1056**	<8	<20	23.56
**K1074**	<8	640	87.45
**K1079**	<8	80	56.35
**K1080**	<8	80	54.74
**K1084**	<8	80	42.18
**K1099**	<8	<20	15.53
**K1103**	<8	<20	24.37
**K1297**	<8	<20	20.67

Clinical human serum samples	Virus neutralization titer	EB-ELISA titer at 30% cut-off	Inhibition in EB-ELISA at 1∶20 dilution
**18411**	<8	<20	18.74
**18425**	<8	<20	13.51
**18482**	<8	<20	13.98
**18574**	<8	<20	13.52
**18588**	<8	<20	9.47
**18605**	<8	<20	14.24
**18608**	<8	<20	12.67
**18666**	<8	<20	16.32

**A. Human serum samples with positive titer in virus neutralization. B. Human serum samples with negative titer in virus neutralization. C. Human cord blood samples.**

### Prevalence evaluation of EV71 antibody

**Figure 6:**
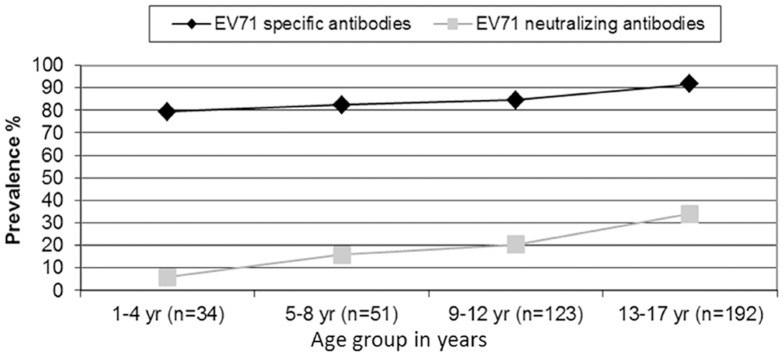
Prevalence (%) of EV71-specific and neutralizing antibody by age group. The prevalence of EV71- specific and neutralizing antibody was evaluated among four age groups. The prevalence of EV71-specific antibody was determined based on the percentage of samples which were positive in EB-ELISA. The prevalence of EV71 neutralizing antibody was determined based on the percentage of samples which were positive in virus neutralization. n: number of samples in each age group.

All the 400 human serum samples were collected from children and adolescents aged 1–17 years in Singapore. Patients with a history of HFMD were excluded to avoid bias in the data. Prevalence of EV71 antibody by age group was summarized in Fig. 6. The positive percentages in both virus neutralization and EB-ELISA increased from younger age groups to older ones. In all four age groups, the EB-ELISA detected a higher positive percentage than virus neutralization. The data from EB-ELISA indicated the prevalence of EV71-specific antibody in different age groups while virus neutralization displayed the prevalence of EV71 neutralizing antibody by age group.

## Discussion

Successful early control of EV71 enterovirus requires active serological surveillance in humans. Despite the availability of conventional methods such as microneutralization assay for serological surveillance, the need for a sensitive and specific serological assay for detection of human antibody against EV71 has not been met. In this study, an EB-ELISA for detection of EV71 antibody was developed based on a Mab 1C6 that recognizes a unique epitope in intact virus. Offering considerable advantages in viral antibody detection, epitope blocking ELISA has been used for sensitive and specific detection of antibodies to many other viral pathogens [37], [38], including H5N1 influenza virus [39].

1C6 recognizes a non-neutralizing conformational epitope in EV71. 1C6 can efficiently detect EV71 expression in infected RD and Vero cells. However, no signal was detected with 1C6 in any recombinant construct expressing single EV71 protein. Interestingly, 1C6 successfully reacted with baculovirus expressing EV71 VLPs, which compose VP0, VP1 and VP3, both in IFA and ELISA. Taken together, these findings indicated that the Mab 1C6 targets a conformation-dependent epitope [40], which exists in EV71 virions with intact native structure [41]. This epitope may be located in a joint binding region of two or more capsid proteins. It is formed with residues distributed in different capsid proteins. Therefore, 1C6 can recognize the epitope either when these residues gather in the correct manner or when those viral capsid proteins involved are assembled into a correct viral structure. This unique property makes 1C6 an antibody to detect EV71 virus or any EV71 capsid protein combination with correct structure, instead of other recombinant EV71 proteins. As this antigenic epitope relies on correct protein folding, it does not exist in any other enteroviruses and it is difficult to mimic this epitope expression in an *in vitro* system in contrast to a linear epitope. These features confer Mab 1C6 with the significant specificity against EV71 without any cross-reactivity to coxsackievirus. Since the epitope is found in naturally occurring EV71 viruses, antibodies targeting it will be elicited in humans exposed to and infected with EV71. 1C6 specifically competes with those antibodies targeting the same epitope in EV71 virus, allowing the development of blocking ELISA with 1C6. Besides, based on the specific reactivity to EV71 virion, 1C6 can serve as a useful tool to detect EV71 antigen accurately. Further identification and characterization of the epitope of 1C6 will throw light on the antigenic structure as well as the virion assembly of EV71.

The absence of EV71 specific antibodies in serum suggests the susceptibility to EV71 virus infection [42]. However, children above age 10 and adults do not develop significant symptom even if they are infected with EV71. As a group of virus carriers, they pose a serious threat on susceptible population of HFMD. Therefore, the specific and sensitive detection of EV71 antibody will be a very effective means for the identification of these different populations. With the increased sensitivity, the results from the 1C6 based blocking ELISA revealed a larger population carrying EV71 antibodies than previously indicated by virus neutralization. This blocking ELISA will help to identify these potential infected adults without symptom, vulnerable individuals as well as other patients infected with EV71 at the early stage. With human cord blood samples as negative controls in the test, no cross reactivity was detected among these human samples of cord blood, confirming the existence of EV71 specific antibodies in those ELISA-positive but VN-negative samples. About 83% of the human serum samples with negative neutralization titers were shown to be positive with the blocking ELISA. This result corresponds to the findings of other research groups. A study performed in Vietnam population found that in children between 5 to 15 years of age, seroprevalence of EV71 neutralizing antibodies was 84%, while the seroprevalence at 1 year of age was 8.3%, at 18 months of age it was 13.4% and by 2 yrs of age, 23.6% [43]. Previous studies from European scientists indicated that the seroprevalence based on virus neutralization for individuals >1 year was found overall to be 42.8% for EV71 in Germany [44]. These findings suggest that majority of young children and infants are susceptible to EV71 infection due to the lack of the EV71 neutralizing antibody, while most adults and teenagers with specific antibodies were exposed to EV71 infection, carrying the pathogen as a potential threat to the younger population. Therefore, the EB ELISA developed in this study will serve as an improved diagnostic method to study serological distribution of EV71 among population by virtue of its higher sensitivity than virus neutralization, thus offering a useful tool in disease prevention and control.

Compared to other serology tests, EB ELISA shows advantages in sensitivity, specificity and ease in processing. No special secondary antibody is required to process any serum samples from any species. This makes the test available to immediate use for human and any animal samples and saves much cost for those expensive secondary antibodies. According to these findings, the EB ELISA can detect fewer amounts of EV71-specific antibodies than virus neutralization, differentiating precisely the carriers of EV71 antibodies from the susceptible population without antibodies. As a highly sensitive test, this blocking ELISA based on 1C6 antibody retains its specificity. This EB ELISA has no cross-reactivity against sera from any animal immunized with various coxsackieviruses. Even though IFA of EV71 infected cells is a sensitive serology test, it cannot differentiate [45] among different enterovirus samples and lacks a sharp cut-off point. In summary, the blocking ELISA with Mab 1C6 developed in this study provides highly sensitive and specific detection of EV71 antibodies and could be formatted into a rapid field test based on dipstick or lateral flow technologies, which would greatly facilitate clinical investigation. Effective control of EV71 in communities of young children is critical as it limits the outbreak of HFMD in this vulnerable population. Rapid serology diagnostic kits as such are inexpensive and easy to perform which would be very beneficial for Asia-Pacific region, where a lot of developing countries are facing endemic EV71 circulation. Therefore, the 1C6 EB ELISA is very attractive option for detection of antibodies to EV71 in human sera for sero-diagnosis and surveillance.

## References

[1] AbuBakar S, Sam IC, Yusof J, Lim MK, Misbah S, et al (2009) Enterovirus 71 outbreak, Brunei. Emerg Infect Dis 15: 79–82. 19116058 10.3201/eid1501.080264 PMC2660687

[2] Schmidt NJ, Lennette EH, Ho HH (1974) An apparently new enterovirus isolated from patients with disease of the central nervous system. J Infect Dis 129: 304–309. 4361245 10.1093/infdis/129.3.304

[3] Hagiwara A, Tagaya I, Yoneyama T (1978) Epidemic of hand, foot and mouth disease associated with enterovirus 71 infection. Intervirology 9: 60–63. 202573 10.1159/000148922

[4] Blomberg J, Lycke E, Ahlfors K, Johnsson T, Wolontis S, et al (1974) Letter: New enterovirus type associated with epidemic of aseptic meningitis and-or hand, foot, and mouth disease. Lancet 2: 112. 10.1016/s0140-6736(74)91684-5 4136956

[5] Kennett ML, Birch CJ, Lewis FA, Yung AP, Locarnini SA, et al (1974) Enterovirus type 71 infection in Melbourne. Bull World Health Organ 51: 609–615. 4377551 PMC2366271

[6] Chumakov M, Voroshilova M, Shindarov L, Lavrova I, Gracheva L, et al (1979) Enterovirus 71 isolated from cases of epidemic poliomyelitis-like disease in Bulgaria. Arch Virol 60: 329–340. 228639 10.1007/BF01317504

[7] Nagy G, Takatsy S, Kukan E, Mihaly I, Domok I (1982) Virological diagnosis of enterovirus type 71 infections: experiences gained during an epidemic of acute CNS diseases in Hungary in 1978. Arch Virol 71: 217–227. 6285858 10.1007/BF01314873

[8] Samuda GM, Chang WK, Yeung CY, Tang PS (1987) Monoplegia caused by Enterovirus 71: an outbreak in Hong Kong. Pediatr Infect Dis J 6: 206–208. 3562141 10.1097/00006454-198702000-00013

[9] Bible JM, Iturriza-Gomara M, Megson B, Brown D, Pantelidis P, et al (2008) Molecular epidemiology of human enterovirus 71 in the United Kingdom from 1998 to 2006. J Clin Microbiol 46: 3192–3200. 18650362 10.1128/JCM.00628-08 PMC2566106

[10] Lum LC, Wong KT, Lam SK, Chua KB, Goh AY, et al (1998) Fatal enterovirus 71 encephalomyelitis. J Pediatr 133: 795–798. 9842048 10.1016/s0022-3476(98)70155-6

[11] Liu CC, Tseng HW, Wang SM, Wang JR, Su IJ (2000) An outbreak of enterovirus 71 infection in Taiwan, 1998: epidemiologic and clinical manifestations. J Clin Virol 17: 23–30. 10814935 10.1016/s1386-6532(00)00068-8

[12] Chan LG, Parashar UD, Lye MS, Ong FG, Zaki SR, et al (2000) Deaths of children during an outbreak of hand, foot, and mouth disease in sarawak, malaysia: clinical and pathological characteristics of the disease. For the Outbreak Study Group. Clin Infect Dis 31: 678–683. 11017815 10.1086/314032

[13] Chang LY (2008) Enterovirus 71 in Taiwan. Pediatr Neonatol 49: 103–112. 19054914 10.1016/S1875-9572(08)60023-6

[14] Singh S, Chow VT, Phoon MC, Chan KP, Poh CL (2002) Direct detection of enterovirus 71 (EV71) in clinical specimens from a hand, foot, and mouth disease outbreak in Singapore by reverse transcription-PCR with universal enterovirus and EV71-specific primers. J Clin Microbiol 40: 2823–2827. 12149336 10.1128/JCM.40.8.2823-2827.2002 PMC120643

[15] van der Sanden S, Koopmans M, Uslu G, van der Avoort H (2009) Epidemiology of enterovirus 71 in the Netherlands, 1963 to 2008. J Clin Microbiol 47: 2826–2833. 19625480 10.1128/JCM.00507-09 PMC2738086

[16] Bible JM, Pantelidis P, Chan PK, Tong CY (2007) Genetic evolution of enterovirus 71: epidemiological and pathological implications. Rev Med Virol 17: 371–379. 17487831 10.1002/rmv.538

[17] McMinn PC (2002) An overview of the evolution of enterovirus 71 and its clinical and public health significance. FEMS Microbiol Rev 26: 91–107. 12007645 10.1111/j.1574-6976.2002.tb00601.x

[18] Qiu J (2008) Enterovirus 71 infection: a new threat to global public health? Lancet Neurol 7: 868–869. 18848307 10.1016/S1474-4422(08)70207-2 PMC7128195

[19] Wu Y, Yeo A, Phoon MC, Tan EL, Poh CL, et al (2010) The largest outbreak of hand; foot and mouth disease in Singapore in 2008: the role of enterovirus 71 and coxsackievirus A strains. Int J Infect Dis 14: e1076–1081. 20952237 10.1016/j.ijid.2010.07.006

[20] Liu CC, Guo MS, Lin FH, Hsiao KN, Chang KH, et al (2011) Purification and characterization of enterovirus 71 viral particles produced from vero cells grown in a serum-free microcarrier bioreactor system. PLoS One 6: e20005. 21603631 10.1371/journal.pone.0020005 PMC3094384

[21] Ranganathan S, Singh S, Poh CL, Chow VT (2002) The hand, foot and mouth disease virus capsid: sequence analysis and prediction of antigenic sites from homology modelling. Appl Bioinformatics 1: 43–52. 15130856

[22] Hogle JM, Chow M, Filman DJ (1985) Three-dimensional structure of poliovirus at 2.9 A resolution. Science 229: 1358–1365. 2994218 10.1126/science.2994218

[23] Takeda N, Tanimura M, Miyamura K (1994) Molecular evolution of the major capsid protein VP1 of enterovirus 70. J Virol 68: 854–862. 8289388 10.1128/jvi.68.2.854-862.1994 PMC236522

[24] Tee KK, Lam TT, Chan YF, Bible JM, Kamarulzaman A, et al (2010) Evolutionary genetics of human enterovirus 71: origin, population dynamics, natural selection, and seasonal periodicity of the VP1 gene. J Virol 84: 3339–3350. 20089660 10.1128/JVI.01019-09 PMC2838098

[25] Minor PD, Ferguson M, Evans DM, Almond JW, Icenogle JP (1986) Antigenic structure of polioviruses of serotypes 1, 2 and 3. J Gen Virol 67 (Pt 7) 1283–1291. 2425046 10.1099/0022-1317-67-7-1283

[26] Fiore L, Ridolfi B, Genovese D, Buttinelli G, Lucioli S, et al (1997) Poliovirus Sabin type 1 neutralization epitopes recognized by immunoglobulin A monoclonal antibodies. J Virol 71: 6905–6912. 9261417 10.1128/jvi.71.9.6905-6912.1997 PMC191973

[27] Buttinelli G, Donati V, Ruggeri FM, Joki-Korpela P, Hyypia T, et al (2003) Antigenic sites of coxsackie A9 virus inducing neutralizing monoclonal antibodies protective in mice. Virology 312: 74–83. 12890622 10.1016/s0042-6822(03)00182-x

[28] Frimann TH, Barfoed AM, Aasted B, Kamstrup S (2007) Vaccination of mice with plasmids expressing processed capsid protein of foot-and-mouth disease virus–importance of dominant and subdominant epitopes for antigenicity and protection. Vaccine 25: 6191–6200. 17640782 10.1016/j.vaccine.2007.06.002

[29] Joki-Korpela P, Roivainen M, Lankinen H, Poyry T, Hyypia T (2000) Antigenic properties of human parechovirus 1. J Gen Virol 81: 1709–1718. 10859376 10.1099/0022-1317-81-7-1709

[30] Alho A, Marttila J, Ilonen J, Hyypia T (2003) Diagnostic potential of parechovirus capsid proteins. J Clin Microbiol 41: 2294–2299. 12791839 10.1128/JCM.41.6.2294-2299.2003 PMC156510

[31] Abed Y, Wolf D, Dagan R, Boivin G (2007) Development of a serological assay based on a synthetic peptide selected from the VP0 capsid protein for detection of human parechoviruses. J Clin Microbiol 45: 2037–2039. 17442804 10.1128/JCM.02432-06 PMC1933058

[32] Petric M, Comanor L, Petti CA (2006) Role of the laboratory in diagnosis of influenza during seasonal epidemics and potential pandemics. J Infect Dis 194 Suppl 2: S98–110. 17163396 10.1086/507554

[33] Stelzer-Braid S, Wong B, Robertson P, Lynch GW, Laurie K, et al (2008) A commercial ELISA detects high levels of human H5 antibody but cross-reacts with influenza A antibodies. J Clin Virol 43: 241–243. 18675584 10.1016/j.jcv.2008.06.012

[34] Meng T, Kolpe AB, Kiener TK, Chow VT, Kwang J (2011) Display of VP1 on the surface of baculovirus and its immunogenicity against heterologous human enterovirus 71 strains in mice. PLoS One 6: e21757. 21747954 10.1371/journal.pone.0021757 PMC3128602

[35] Yokoyama WM, Christensen M, Santos GD, Miller D (2006) Production of monoclonal antibodies. Curr Protoc Immunol Chapter 2: Unit 2 5. 10.1002/0471142735.im0205s74 18432969

[36] Lim XF, Jia Q, Khong WX, Yan B, Premanand B, et al (2012) Characterization of an isotype-dependent monoclonal antibody against linear neutralizing epitope effective for prophylaxis of enterovirus 71 infection. PLoS One 7: e29751. 22279543 10.1371/journal.pone.0029751 PMC3261156

[37] Singh BK, Ahuja S, Gulati BR (2004) Development of a neutralizing monoclonal antibody-based blocking ELISA for detection of equine herpesvirus 1 antibodies. Vet Res Commun 28: 437–446. 15379438 10.1023/b:verc.0000034996.18533.90

[38] Kuck D, Kern A, Kleinschmidt JA (2007) Development of AAV serotype-specific ELISAs using novel monoclonal antibodies. J Virol Methods 140: 17–24. 17126418 10.1016/j.jviromet.2006.10.005

[39] Prabakaran M, Ho HT, Prabhu N, Velumani S, Szyporta M, et al (2009) Development of epitope-blocking ELISA for universal detection of antibodies to human H5N1 influenza viruses. PLoS One 4: e4566. 19238211 10.1371/journal.pone.0004566 PMC2642733

[40] Olcese VA, Chen Y, Schlegel R, Yuan H (2004) Characterization of HPV16 L1 loop domains in the formation of a type-specific, conformational epitope. BMC Microbiol 4: 29. 15260888 10.1186/1471-2180-4-29 PMC499545

[41] Yamaguchi T, Iwata K, Kobayashi M, Ogawa M, Fukushi H, et al (1996) Epitope mapping of capsid proteins VP2 and VP3 of infectious bursal disease virus. Arch Virol 141: 1493–1507. 8856029 10.1007/BF01718250

[42] Ang LW, Phoon MC, Wu Y, Cutter J, James L, et al (2011) The changing seroepidemiology of enterovirus 71 infection among children and adolescents in Singapore. BMC Infect Dis 11: 270. 21988931 10.1186/1471-2334-11-270 PMC3198955

[43] Tran CB, Nguyen HT, Phan HT, Tran NV, Wills B, et al (2011) The seroprevalence and seroincidence of enterovirus71 infection in infants and children in Ho Chi Minh City, Viet Nam. PLoS One 6: e21116. 21765891 10.1371/journal.pone.0021116 PMC3134465

[44] Rabenau HF, Richter M, Doerr HW (2010) Hand, foot and mouth disease: seroprevalence of Coxsackie A16 and Enterovirus 71 in Germany. Med Microbiol Immunol 199: 45–51. 19941005 10.1007/s00430-009-0133-6

[45] Gallagher S, Winston SE, Fuller SA, Hurrell JG (2004) Immunoblotting and immunodetection. Curr Protoc Neurosci Chapter 5: Unit 5 19. 10.1002/0471142301.ns0519s29 18428601

